# Criticality Analysis of the Lower Ionosphere Perturbations Prior to the 2016 Kumamoto (Japan) Earthquakes as Based on VLF Electromagnetic Wave Propagation Data Observed at Multiple Stations

**DOI:** 10.3390/e20030199

**Published:** 2018-03-16

**Authors:** Stelios M. Potirakis, Tomokazu Asano, Masashi Hayakawa

**Affiliations:** 1 Department of Electrical and Electronics Engineering, University of West Attica, Campus 2, 250 Thivon and P. Ralli, Aigaleo, Athens, GR-12244, Greece; 2 Hayakawa Institute of Seismo Electromagnetics Co. Ltd., University of Electro-Communications (UEC), Alliance Center #521, 1-1-1 Kojimacho, Chofu, Tokyo 182-0026, Japan

**Keywords:** 2016 Kumamoto EQs, subionospheric VLF/LF propagation, critical dynamics, natural time analysis

## Abstract

The perturbations of the ionosphere which are observed prior to significant earthquakes (EQs) have long been investigated and could be considered promising for short-term EQ prediction. One way to monitor ionospheric perturbations is by studying VLF/LF electromagnetic wave propagation through the lower ionosphere between specific transmitters and receivers. For this purpose, a network of eight receivers has been deployed throughout Japan which receive subionospheric signals from different transmitters located both in the same and other countries. In this study we analyze, in terms of the recently proposed natural time analysis, the data recorded by the above-mentioned network prior to the catastrophic 2016 Kumamoto fault-type EQs, which were as huge as the former 1995 Kobe EQ. These EQs occurred within a two-day period (14 April: $M_{W}=6.2$ and $M_{W}=6.0$, 15 April: $M_{W}=7.0$) at shallow depths (~10 km), while their epicenters were adjacent. Our results show that lower ionospheric perturbations present critical dynamics from two weeks up to two days before the main shock occurrence. The results are compared to those by the conventional nighttime fluctuation method obtained for the same dataset and exhibit consistency. Finally, the temporal evolutions of criticality in ionospheric parameters and those in the lithosphere as seen from the ULF electromagnetic emissions are discussed in the context of the lithosphere-atmosphere-ionosphere coupling.

## 1. Introduction

In a large number of relevant articles published during the last few decades a variety of electromagnetic (EM) phenomena have been reported to appear prior to an earthquake (EQ), e.g., [1,2,3,4,5,6,7,8]. Specifically, the ionosphere has statistically been confirmed to be correlated with EQs in such a way that it presents remarkable sensitivity to EQ preparation processes happening in the lithosphere, e.g., [9,10]. A variety of pre-EQ EM phenomena related to different kinds of ionospheric anomalies have long been investigated at different frequency bands using multiple methods and could be considered promising for short-term EQ prediction [4,7,10,11,12,13,14,15,16,17,18,19,20].

The catastrophic 2016 Kumamoto EQs (14–15 April 2016) caused 50 deaths, over 1800 injuries and serious damage to local infrastructures [21]. These were fault-type EQs just like the 1995 Kobe EQ [22] and the magnitude of the main shock was as big as that of the Kobe EQ. In this sense, it is worthwhile to investigate the EM phenomena for these Kumamoto events. A variety of different ionosphere-related precursory signatures possibly related to the Kumamoto EQs have already been reported, such as ionospheric anomalies as detected by total electron content (TEC) calculated by GNSS receivers [23], or GIM-TEC [24], or GPS-TEC [25], transient variations on atmospheric and ionospheric parameters [26,27], subionospheric very low frequency (VLF) propagation anomalies [28,29], atmospheric ULF/ELF radiation and ULF depression [30], criticality in the ground-observed ULF magnetic fields [31], and intermittency-induced criticality in the VLF subionospheric propagation data [32].

We focus here on the lower ionosphere perturbations as monitored by the VLF propagation anomalies. A network of eight VLF/LF receivers has been operating during the last few years throughout Japan which receive subionospheric signals from different transmitters located both in the same and other countries. In this study we perform a criticality analysis of the data acquired by the specific network prior to the 2016 Kumamoto EQs. The analysis is performed by means of the recently proposed natural time (NT) method [33,34,35]. Beyond the seismic electric signals (SES) of Varotsos’ group, the NT method has already been successfully applied on other EM variations possibly related to EQs, such as MHz-kHz EM emissions [8,36,37] and ground-observed ULF magnetic fields (e.g., [31,38,39,40]). Yet, this is the first attempt of application of NT analysis to VLF subionospheric propagation data.

The way of application of the NT analysis is initially investigated, as well as its sensitivity to other possible causes of lower ionosphere anomalies. Then, the data from all eight stations of the aforementioned network of VLF/LF receivers concerning their receptions from a specific transmitter located in south Japan (with call sign “JJI”) are analyzed. The NT analysis results reveal that the lower ionosphere presented characteristics of critical dynamics from two weeks up to two days before the main shock. Moreover, as compared to the results obtained by analysis of the same data with the conventional nighttime fluctuation method and wave-hop theoretical computations [28,29] a remarkable consistency is observed.

The remaining of the article is organized as follows: Section 2 provides EQ information and describes the network of VLF/LF stations used in this study, as well as the data to be analyzed; a brief description of the key concepts and basic formulas of the NT analysis method is provided in Section 3; the NT analysis of the VLF subionospheric propagation data is presented in Section 4, along with a discussion on the sensitivity of the method to other causes of ionospheric anomalies. In Section 5, the results obtained in Section 4 are compared with the findings of the conventional nighttime fluctuation method, while the findings of NT analysis applied to the ULF electromagnetic emissions of the same period are discussed within the context of lithosphere-atmosphere-ionosphere (LAI) coupling; the global geomagnetic activity during the analyzed time period is also discussed. Finally, the main findings are summarized in Section 6.

## 2. EQs Information, VLF/LF Stations Network, Subionospheric Propagation Data

**Figure 1:**
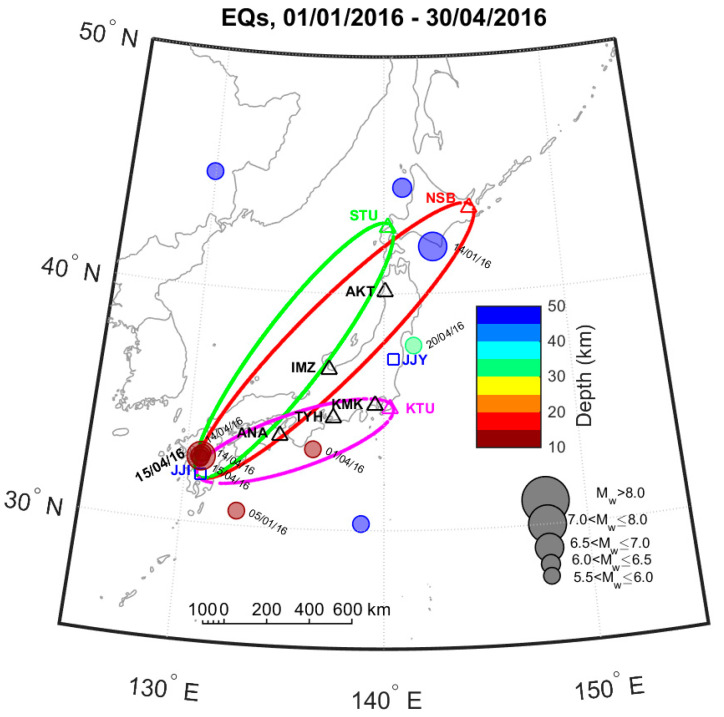
Map of the wider area of Japan, showing the network of VLF/LF receivers (triangles), the VLF transmitters (rectangles), the 5th Fresnel zones for the JJI-NSB, JJI-STU and JJI-KTU paths, as well as all EQs with $M_{W}>5.5$ which happened during the time period from 1 January 2016 to 30 April 2016. The circle size is proportional to EQ’s magnitude and its color refers to hypocenter’s depth. The date of occurrence appears only for the EQs with hypocenter at depth <100 km. The Kumamoto EQs correspond to the large-sized overlapping circles in south-west Japan (on Kyushu Island).

In this article we use VLF subionospheric propagation data between the transmitter “JJI”, located at Miyazaki in south Japan (geographic coordinates: 32.045° N, 130.811° E), shown in Figure 1 as a rectangle, and eight receivers dispersed all around Japan, shown in Figure 1 as triangles; the abbreviations of all receivers and all monitored transmitters along with their transmission signal frequencies are summarized in Table 1. Figure 1 also shows the 5th Fresnel zones only for three indicative links, defining an area over which the propagation path is considered sensitive to EQ preparation processes, e.g., [41]. In the same figure, all EQs with $M_{W}>5.5$ which happened in a wide area around Japan are also shown for the time period from 1 January 2016 to 30 April 2016. Even though an extremely low probability of ~1% was expected by the medium-term EQ prediction in the region of Kumamoto, a series of large magnitude EQs occurred, with a main event as big as the 1995 Kobe EQ. The three fault-type recent EQs of our interest happened in south-west Japan at a very close epicentral distance under the city of Kumamoto in the following order [42]: (1) $M_{W}=6.2$, 14 April 2016, 12:26:41.1 UT (32.788° N, 130.704° E), depth ~ 9 km; (2) $M_{W}=6.0$, 14 April 2016, 15:03:50.6 UT (32.697° N, 130.720° E), depth ~ 8 km; (3) $M_{W}=7.0$, 15 April 2016, 16:25:15.7 UT (32.791° N, 130.754° E), depth ~ 10 km.

In this study we use the same data which are employed by the conventional nighttime fluctuation method [12]. Specifically, from the original raw measurements (sampling frequency $f_{s}=1\;\mathrm{Hz}$) we first calculate the residue dA(t) between the received signal amplitude A(t) and an average signal amplitude 〈A(t)〉 calculated by means of a running average over ±15 days as dA(t)=A(t)−〈A(t)〉. Then, since the (local) daytime data have been observed to exhibit too small disturbances to be analyzed, we use only (local) nighttime data over four time periods around the year (10:00–20:00 UT for 22/11–21/02, 11:00–19:00 UT for 22/02–21/05, 11:30–17:30 for 22/05–21/09, 10:30–19:00 for 22/09–21/11) to calculate daily values (1 value/day) for three quantities TR (“trend”), DP (“dispersion”), and NF (“nighttime fluctuation”):

(1) $$TR=\frac{\sum_{N_{s}}^{N_{e}}dA\left(t\right)}{N_{e}-N_{s}},$$

which is actually the mean value of dA(t), where $N_{s}$ and $N_{e}$ are the time points of the start and end of the above defined nighttime periods,

(2) $$DP=\sqrt{\frac{1}{N_{e}-N_{s}}\sum_{N_{s}}^{N_{e}}{\left(dA\left(t\right)-TR\right)}^{2}}$$

which is actually the standard deviation of dA(t), and

(3) $$NF=\sum_{N_{s}}^{N_{e}}{\left(dA\left(t\right)\right)}^{2}.$$

**Figure 2:**
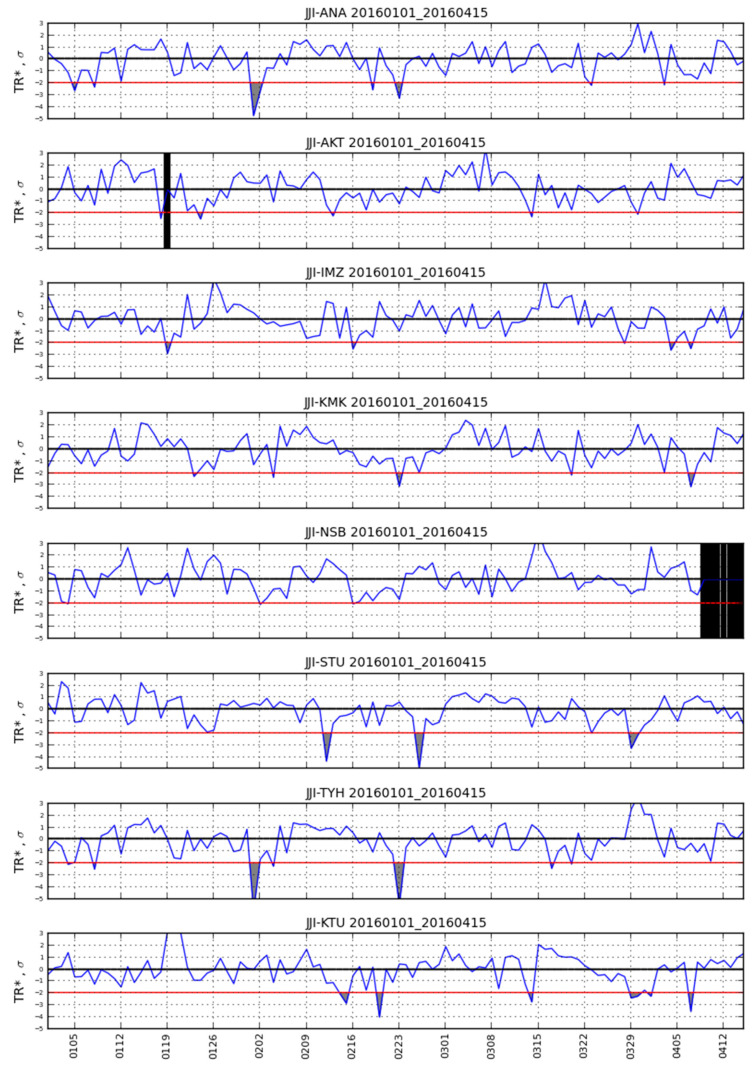
VLF subionospheric propagation characteristic quantity TR* during the time period 1 January 2016–15 April 2016 for the receptions of the eight receivers from the transmitter JJI (see also Table 1 and Figure 1). Values exceeding −2*σ* threshold (shown as red horizontal lines) are considered as statistically significant anomalies (grey-color filled areas) according to the conventional nighttime fluctuation method [12]. Black vertical patches indicate time periods for which data are missing due to any reason. The horizontal time (*x*-) axis shows date (UT).

Note that in the conventional nighttime fluctuation method, the normalized values of the above quantities, denoted respectively as DP*, TR*, and NF*, are usually studied. These are calculated as $X*=\left(X-{\langle X\rangle }_{\pm 15days}\right)/\sigma_{\pm 15days}$ where ${\langle X\rangle }_{\pm 15days}$ and $\sigma_{\pm 15days}$ denote the mean value and standard deviation ±15 days around the day of interest, respectively. However, in this paper we use the non-normalized values of DP, TR, and NF as defined in Equations (1)–(3). As an example of the observed VLF subionospheric propagation variations, Figure 2 shows TR* variation during the time period 1 January 2016–15 April 2016 for the receptions of the eight receivers from the transmitter JJI. We should clarify that the conventional nighttime fluctuation method is not employed in this paper; however, the already published conventional nighttime fluctuation method results related to the 2016 Kumamoto EQs [28,29] are discussed in the discussion section (see Section 5), in comparison with the herein presented results (see Section 4) which are obtained by the NT analysis method.

## 3. Natural Time Analysis Method

The natural time (NT) analysis method was originally proposed for the analysis of ultra-low frequency (≤1 Hz) SES signals [33,34,43], and has been shown to be optimal for enhancing the signals in the time-frequency space [44]. The transformation of a time series of “events” from the conventional time domain to the NT domain is performed by ignoring the time-stamp of each event and retaining only their normalized order (index) of occurrence. Explicitly, in a time series of *N* successive events, the NT, $\chi_{k}$, of the kth event is the index of occurrence of this event normalized by dividing by the total number of the considered events, $\chi_{k}=k/N$. On the other hand, the “energy”, $Q_{k}$ of each kth event is preserved. We note that the quantity $Q_{k}$ represents different physical quantities for various time series: for EQ time series it has been assigned to a seismic energy released (e.g., seismic moment) [34], and for SES signals that are of dichotomous nature it corresponds to SES pulse duration [34], while for geophysical scale MHz EM emission signals that are of non-dichotomous nature, it has been attributed to the energy of fracto-electromagnetic emission events as defined in [36]. The transformed time series ($\chi_{k}$, $Q_{k}$) is then studied through the normalized power spectrum $\Pi \left(\varpi \right)={\left|\sum_{k=1}^{N}p_{k}\exp\left(j\varpi \chi_{k}\right)\right|}^{2}$, where ϖ is the natural angular frequency, ϖ=2πφ, with φ standing for the frequency in NT, termed “natural frequency”, and $p_{k}=Q_{k}/\sum_{n=1}^{N}Q_{n}$ corresponds to the kth event’s normalized energy. Note that the term “natural frequency” should not be confused with the rate at which a system oscillates when it is not driven by an external force; it defines an analysis domain dual to the NT domain, in the framework of Fourier–Stieltjes transform [35].

The study of Π(ϖ) at ϖ close to zero reveals the dynamic evolution of the time series under analysis. This is because all the moments of the distribution of $p_{k}$ can be estimated from Π(ϖ) at ϖ→0 [45]. Aiming to that, by the Taylor expansion $\Pi \left(\varpi \right)=1-\kappa_{1}\varpi^{2}+\kappa_{2}\varpi^{4}+\ldots$ the quantity $\kappa_{1}$ is defined, where $\kappa_{1}=\sum_{k=1}^{N}p_{k}\chi_{k}^{2}-{\left(\sum_{k=1}^{N}p_{k}\chi_{k}\right)}^{2}$, i.e., the variance of $\chi_{k}$ weighted for $p_{k}$ characterizing the dispersion of the most significant events within the “rescaled” interval (0, 1]. Moreover, the entropy in NT, $S_{nt}$, is defined [46] as $S_{nt}=\sum_{k=1}^{N}p_{k}\chi_{k}\ln\chi_{k}-\left(\sum_{k=1}^{N}p_{k}\chi_{k}\right)\ln\left(\sum_{k=1}^{N}p_{k}\chi_{k}\right)$ and corresponds [35,46] to the value at q=1 of the derivative of the fluctuation function $fl\left(q\right)=\langle \chi^{q}\rangle -{\langle \chi \rangle }^{q}$ with respect to q (while $\kappa_{1}$ corresponds to fl(q) for q=2). It is a dynamic entropy depending on the sequential order of events [46]. The entropy, $S_{nt-}$, obtained upon considering [46] the time reversal T, i.e., $Tp_{m}=p_{N-m+1}$, is also taken into account.

A system is considered to approach criticality when the parameter $\kappa_{1}$ converges to the value $\kappa_{1}=0.070$ and at the same time both the entropy in NT and the entropy under time reversal satisfy the condition $S_{nt},S_{nt-}<S_{u}=\left(\ln2/2\right)-1/4$ [47], where $S_{u}$ stands for the entropy of a “uniform” distribution in NT [46]. It has to be mentioned that the criterion of the $\kappa_{1}=0.070$ value has originally been derived for SES activity and later on the basis of the Ising model. Its validity has been confirmed on real SES time series, while it has also been verified to be valid for several self-organized criticality (SOC) models and real time series of a variety of applications. In all these dynamical systems, it has been found that the value $\kappa_{1}=0.070$ can be considered as quantifying the extent of the organization of the system at the onset of the critical stage [35].

In the special case of NT analysis of foreshock seismicity [34,43,46,48], the seismicity is considered to be in a true critical state, a “true coincidence” is achieved, when three additional conditions are satisfied: (i) The “average” distance 〈D〉 between the curves of normalized power spectra Π(ϖ) of the evolving seismicity and the theoretical estimation of Π(ϖ), $\Pi_{critical}\left(\varpi \right)=\left(18/5\varpi^{2}\right)-\left(6\cos\varpi /5\varpi^{2}\right)-\left(12\sin\varpi /5\varpi^{3}\right),$ $\Pi_{critical}\left(\varpi \right)\approx 1-\kappa_{1}\varpi^{2},$ for $\kappa_{1}=0.070$ should be smaller than $10^{-2}$, i.e., $\langle D\rangle =\langle \left|\Pi \left(\varpi \right)-\Pi_{critical}\left(\varpi \right)\right|\rangle <10^{-2}$ (this is a practical criterion for signaling the achievement of spectral coincidence) [35]; (ii) the parameter $\kappa_{1}$ should approach the value $\kappa_{1}=0.070$ “by descending from above”, i.e., before the main event the parameter $\kappa_{1}$ should gradually decrease until it reaches the critical value 0.070 (this rule was found empirically) [35,43]; (iii) Since the underlying process is expected to be self-similar, the time of the true coincidence should not vary upon changing (within reasonable limits) either the magnitude threshold, $M_{thres}$, or the area, used in the calculation.

It should be finally clarified that in the case of seismicity analysis, the temporal evolution of the parameters $\kappa_{1}$, $S_{nt}$, $S_{nt-}$, and 〈D〉 is studied as new events that exceed the magnitude threshold $M_{thres}$ are progressively included in the analysis. Specifically, as soon as one more event is included, first the time series ($\chi_{k}$, $Q_{k}$) is rescaled in the NT domain, since each time the kth event corresponds to a NT $\chi_{k}=k/N$, where N is the progressively increasing (by each new event inclusion) total number of the considered successive events; then all the parameters involved in the NT analysis are calculated for this new time series; this process continues until the time of occurrence of the main event.

Note that in the case of NT analysis of foreshock seismicity, the introduction of magnitude threshold, $M_{thres}$, excludes some of the weaker EQ events (with magnitude below this threshold) from the NT analysis. On one hand, this is necessary in order to exclude events for which the recorded magnitude is not considered reliable; depending on the installed seismographic network characteristics, a specific magnitude threshold is usually defined to assure data completeness. On the other hand, the use of various magnitude thresholds, $M_{thres}$, offers a means of more accurate determination of the time when criticality is reached. In some cases, it happens that more than one time-points may satisfy the rest of NT critical state conditions, however the time of the true coincidence is finally selected by the last condition that “true coincidence should not vary upon changing (within reasonable limits) either the magnitude threshold, $M_{thres}$, or the area, used in the calculation”.

## 4. Analysis of Lower Ionosphere Nighttime Fluctuations Prior to the 2016 Kumamoto EQs

We apply here the NT method to the nighttime VLF propagation characteristic quantities TR, DP, and NF defined in Section 2, in a similar way to that for the ULF characteristics corresponding to magnetic field variation recorded prior to significant EQs [31,38,39,40]. Specifically: (i) We consider each daily value which is above a certain threshold as an event. In our nighttime VLF propagation characteristic quantities cases (TR, DP, and NF), the “energy” of kth event, that is the value of the quantity $Q_{k}$ of NT analysis (see Section 3), is considered to be equal to the corresponding non-normalized value of each one of the above quantities (as defined by Equations (1)–(3)), provided that this is above a certain threshold such as $TR_{thres}$, $DP_{thres}$, and $NF_{thres}$, respectively; (ii) Then, the NT analysis is performed as in the case of pre-EQ seismic activity on the revealed “events”. Starting from a specific day, all the parameters ($\kappa_{1}$, $S_{nt}$, $S_{nt-}$, 〈D〉 defined in Section 3) are calculated for the time series of events rescaled in the NT domain each time a new event is added, checking for the corresponding criticality criteria as presented in Section 3 for the case of seismicity.

Before applying the NT analysis, the starting time of the analysis has to be determined. Since the ionosphere is known to be sensitive not only to pre-EQ processes, but also to a variety of different kinds of phenomena such as solar flares, magnetic storms, typhoons, tsunamis, and volcano eruptions, e.g., [41,49,50], we have first to check for the sensitivity of the NT analysis to such kind of phenomena. If the NT analysis is sensitive, i.e., if it indicates critical features before such phenomena, these should be either excluded from the analysis period, by setting the start time after their occurrence, or they have to be taken into account during the evaluation of the analysis results. This is because, if the NT analysis intended to examine the possibly EQ-related behavior of the ionosphere starts before a non-EQ-preparation-related phenomenon which undergoes a critical state, this might cause “masking” of the possible critical behavior of the ionosphere due to any EQ preparation processes.

**Figure 3:**
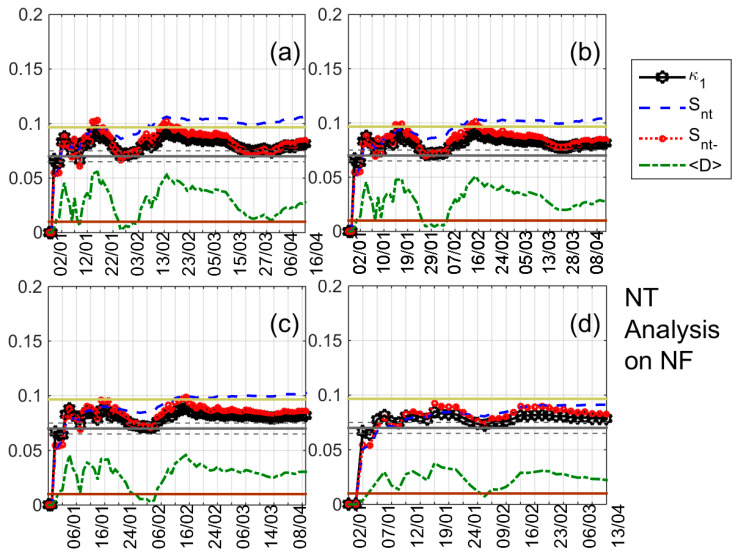
NT analysis of NF for the JJI-IMZ path for the time period 1 January 2016–15 April 2016. Variations of the NT analysis parameters ($\kappa_{1}$, $S_{nt}$, $S_{nt-}$, and 〈D〉) for the different thresholds (**a**) 2, (**b**) 8; (**c**) 12; and (**d**) 20, respectively. The entropy limit of $S_{u}\left(\approx 0.0966\right)$, the $\kappa_{1}$ value 0.070 and a region of ±0.005 around it are shown by the horizontal solid light green, solid grey and the grey dashed lines, respectively, while the 〈D〉 limit (10^−2^) is shown by the horizontal solid brown line. Note that the events employed depend on the considered threshold. Moreover, the time (*x*-) axis (date, UT) is not linear in terms of the conventional time of occurrence of the events, since the employed events appear equally spaced relative to *x*-axis as the NT representation demands, although they are not equally spaced in conventional time.

Knowing that the conventional nighttime fluctuation method indicated a clear pre-EQ anomaly before the 2016 Kumamoto EQs in the JJI-IMZ path [28] (see Table 1 for station abbreviations), as well as the fact that a volcanic eruption occurred in the wider area (Sakurajima Volcano, geographic coordinates: 31°35′ N, 130°39′ E) on 5 February 2016 19:13 JST, we initially tried to apply NT analysis for the time period 1 January 2016–15 April 2016. The results obtained for the specific time period show that the NT analysis of the studied nighttime VLF propagation characteristics is affected by the volcanic eruption focusing on this phenomenon and masking any possible critical behavior due to phenomena which happened after that, including possible EQ-related ones. As an example, part of the NT analysis results for *NF* of the JJI-IMZ path during the time period 1 January 2016–15 April 2016 are shown in Figure 3, indicating that criticality is reached a few days before the abovementioned volcanic eruption. Specifically, according to the NT analysis criticality criteria for the case of seismicity (see Section 3) we observe in Figure 3 that criticality criteria are satisfied on 24 January 2016, i.e., ~2 weeks before the eruption. Therefore, since a disturbance in nighttime VLF propagation characteristics due to any phenomenon can be monitored by observing the fluctuation of their normalized version DP*, TR*, and NF* (see Section 2) it was decided to follow the rule of setting the initial time point for NT analysis at least a few (e.g., ~5) days after the day for which any normalized nighttime VLF propagation characteristic has exceeded the limit of ±2*σ*. According to this rule, it was decided that in order to reveal any possible critical characteristics related to the Kumamoto EQs, the NT analysis should start on 20 March 2016, because on 14 March 2016 there was high variation of the abovementioned normalized characteristics for some of the employed stations [28] (see also Figure 2).

**Figure 4:**
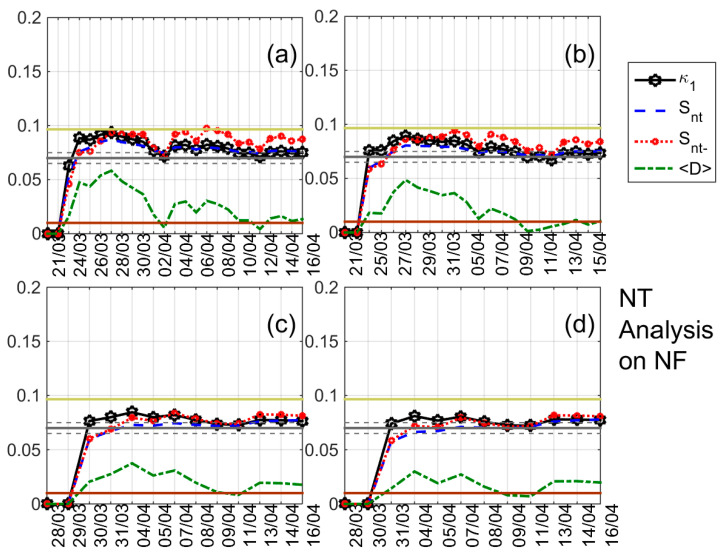
Variations of the NT analysis parameters ($\kappa_{1}$, $S_{nt}$, $S_{nt-}$ and 〈D〉) corresponding to the analysis of *NF* for the JJI-IMZ path during the time period 20 March 2016–15 April 2016 for four threshold values (**a**) 4, (**b**) 7, (**c**) 11 and (**d**) 14, respectively. Figure format is similar to Figure 3.

**Figure 5:**
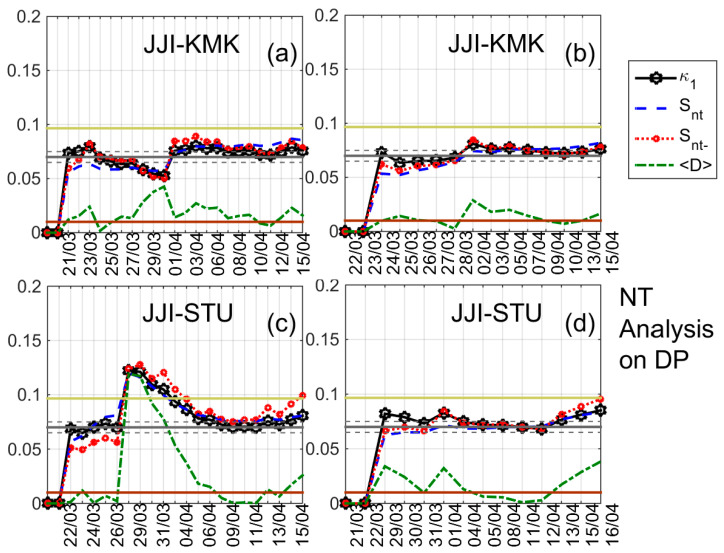
NT analysis of DP; variations of the NT analysis parameters ($\kappa_{1}$, $S_{nt}$, $S_{nt-}$ and 〈D〉); JJI-KMK path, two different thresholds, (**a**) 0.30 and (**b**) 0.72, respectively; JJI-STU path, two different thresholds (**c**) 0.12 and (**d**) 0.54, respectively. Figure format is similar to Figure 3.

**Figure 6:**
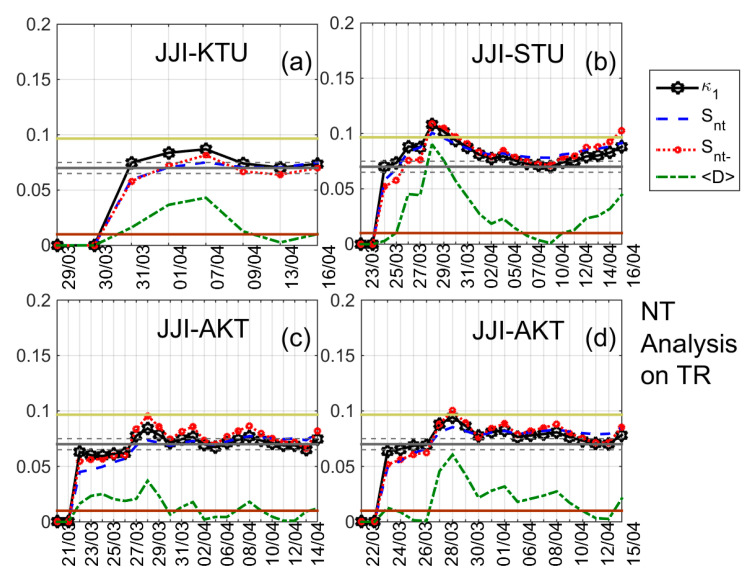
NT analysis of TR; variations of the NT analysis parameters ($\kappa_{1}$, $S_{nt}$, $S_{nt-}$ and 〈D〉); (**a**) an example (threshold = 2.1) of the JJI-KTU path; (**b**) an example (threshold = 1.5) of the JJI-STU path; JJI-AKT path, two different thresholds (**c**) 0.24 and (**d**) 0.48, respectively. Figure format is similar to Figure 3.

**Figure 7:**
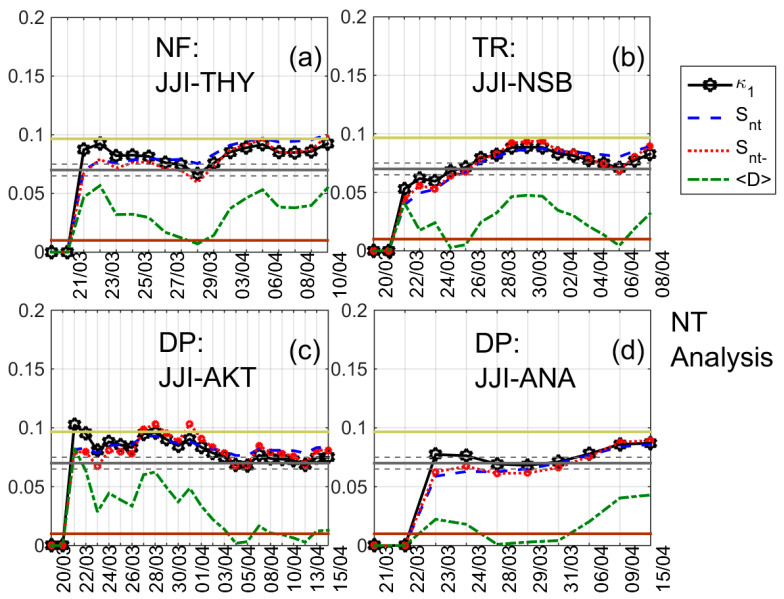
NT analysis examples for cases marginally satisfying criticality criteria; variations of the NT analysis parameters ($\kappa_{1}$, $S_{nt}$, $S_{nt-}$ and 〈D〉). (**a**) *NF* for the JJI-THY path (threshold = 9); (**b**) *TR* for the JJI-NSB path (threshold = 0.33); (**c**) *DP* for the JJI-AKT path (threshold = 0.10); (**d**) *DP* for the JJI-ANA path (threshold = 0.99). Figure format is similar to Figure 3.

The results of the NT analysis of NF, DP, and TR for all eight receiving stations and the time period 20 March 2016–15 April 2016 are summarized in Table 2, while indicative results are shown in Figure 4, Figure 5, Figure 6 and Figure 7. In Figure 4, Figure 5 and Figure 6 clear criticality indications for NF, DP, and TR, respectively, are presented, while Figure 7 portrays examples of marginal criticality indications (please refer to NT analysis criticality criteria for the case of seismicity in Section 3). For example, we can see in Figure 4 that there is a specific time period, namely 11–12 April 2016, during which the NT analysis criticality criteria, i.e., $\kappa_{1}$ reaching the value 0.070 “from above” while at the same time $S_{nt},S_{nt-}<\left(\ln2/2\right)-1/4\;\left(\approx 0.0966\right)$ and $\langle D\rangle <10^{-2}$ are satisfied for the presented four different threshold cases. Therefore, the analysis of NF for the JJI-IMZ path during the time period 20 March 2016–15 April 2016 which is presented in Figure 4 shows clearly that the specific quantity reaches criticality on 11–12 April 2016. As an example of marginal criticality indications, we can refer to the analysis of DP for the JJI-ANA path shown in Figure 7d. In the specific case, we consider that the criterion that the parameter $\kappa_{1}$ should approach the value $\kappa_{1}=0.070$ “by descending from above” is marginally satisfied, since only a few values of $\kappa_{1}$ could be calculated for the specific threshold before it approached the value $\kappa_{1}=0.070$ on 28–30 March 2016. We should clarify that the existence of just one threshold (or very few thresholds) for which criticality conditions are satisfied, and NT analysis parameters ($\kappa_{1}$, $S_{nt}$, $S_{nt-}$, 〈D〉) values which are very close to the limits set by the corresponding criteria, are also considered as “marginal criticality indications”. We observe from Table 2 as follows:

(1) Criticality has been revealed for all eight stations, but for different propagation characteristics (NF, DP, and TR) we have criticality for different combinations of stations and dates.
(2) It is especially worth noting that the receiving stations KMK, TYH, ANA and KTU, which are all situated on the east (Pacific) coast of Japan showed either marginal or clear indications of criticality from 28 March 2016 up to 1–2 April 2016. However, it may be possible that this behavior is related to the M5.9 EQ which happened in the Pacific Ocean coast of Japan on 1 April 2016, 02:39:08.06 UT (33.3835° N, 136.3857° E), depth = 14 km (please also see Figure 1), and not to the 2016 Kumamoto EQs.
(3) After those dates, clear criticality indications are progressively appearing from 5 April 2016 (i.e., 9–10 days before the 2016 Kumamoto EQs) up to 13 April 2016 (i.e., 1–2 days before the 2016 Kumamoto EQs), while marginal indications of criticality are observed even on 15 April 2016 but only at KMK station.
(4) The timeline of clear criticality indications develops as follows: (a) first criticality is found in DP of the JJI-KTU path (5–7 April 2016); (b) then, criticality appears in the JJI-STU path (starting from 8 April 2016 in DP, and TR); (c) between 9 April 2016 and 12–13 April 2016, clear criticality evidence appear in five propagation paths of JJI-STU, JJI-KTU, JJI-IMZ, JJI-KMK, JJI-AKT, some of them presenting critical characteristics in more than one of the analyzed quantities (TR, DP, and NF).

## 5. Discussion

First we try to compare the NT analysis results presented in the previous section with the results by the conventional analysis presented in [28]. As indicated by points (2) and (3) in the previous section, we pay attention to the time period after 5 April because propagation anomalies during this period are more likely to be related with the Kumamoto EQs. In [28] it has been reported that propagation anomalies were observed during the time period 19 March–10 April, which is consistent with the NT analysis results presented in this paper.

A further extensive study has recently been presented in [29]. The temporal evolutions of amplitude changes at all eight receiving stations, have enabled Asano and Hayakawa [29] to compare those with the theoretical estimations with the use of wave-hop method. In the wave-hop method, one can change, independently, the reflection height (lower ionosphere height) (either increasing or decreasing) of 1-hop sky wave and that of 2-hop sky wave close to the transmitter, to estimate the amplitude at all stations. The comparisons between the observed and theoretical amplitudes at all stations allowed them to deduce the spatio-temporal evolution of the ionospheric perturbation associated with the Kumamoto EQs. As a result, the perturbation begins to appear on 3–4 April 2016 (about two weeks before the EQs) and it continues to develop spatially, i.e., horizontal spatial extent is expanding and vertical scale is decreasing (decrease in the lower ionosphere). The maximum of spatial development happens 10–12 April 2016, and is followed by a rapid decay. These spatio-temporal evolutions are found to be in extremely good consistency with the NT analysis findings of criticality in this paper. The dates of maximum spatial development in the ionospheric perturbation are consistent with those in Table 2 indicating when criticality was reached for different propagation parameters of different subionospheric paths.

**Figure 8:**
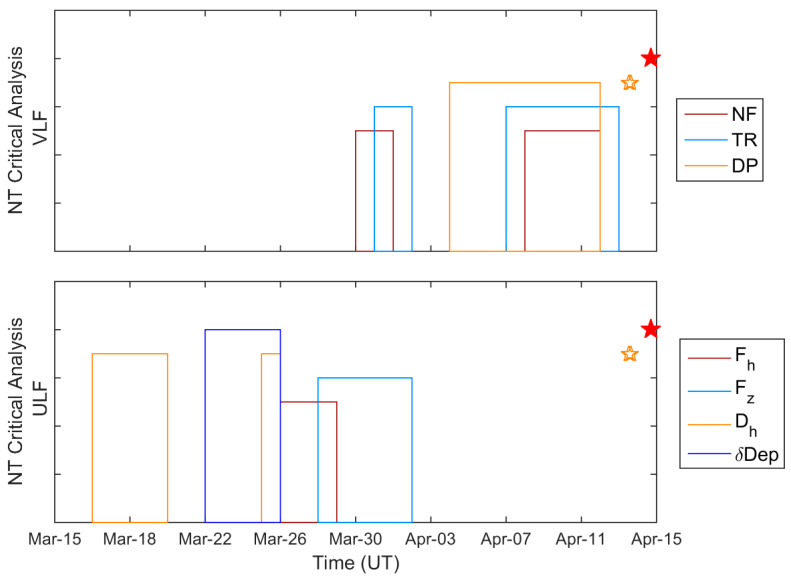
Temporal evolutions of criticality as revealed by NT analysis for the VLF subionospheric propagation parameters NF, TR, and DP (top panel), and for the ULF magnetic field characteristics $F_{h}$, $F_{z}$, $D_{h}$ and δDep (bottom panel). *Y*-axis has no scale or units; only dates of criticality are indicated, while the differences in the *y*-axis dimension just serve as a way for easy visual discrimination of the criticality dates for the different parameters/characteristics, especially for overlapping/adjacent cases. The star symbols indicate the time of occurrence of the Kumamoto EQs.

The generation mechanism of seismo-ionospheric perturbation is poorly understood, but a few hypotheses have already been proposed [50,51,52,53]. The major agent of ionospheric perturbations is likely to be located in the lithosphere or in the near surface. So, it is worthwhile to compare the NT analysis results of this paper with those referring to the lithosphere as seen from the lithospheric ULF electromagnetic emissions as recorded by the ground-based magnetic observatory of Kanoya (KNY) [31] (beyond the time series analysis, full information about the observatory and the involved instrumentation is also provided in [31]). Note that in both cases NT analysis was applied to time series of daily values (1 value/day), representing VLF subionospheric propagation characteristics and ULF magnetic field characteristics, respectively. Figure 8 illustrates a comparison of dates of criticality revealed by the NT analysis method in the ionosphere (top panel) and that in the lithosphere as seen from ULF electromagnetic radiation (bottom panel). This figure indicates that criticality in the lithosphere has been reached about 1 month to 2 weeks before the Kumamoto EQs, whereas criticality can be observed in the ionosphere as VLF propagation anomaly about 2 weeks to a few days before the EQs. So we can find a significant difference in dates of criticality (of the order of 1–2 weeks) in the lithosphere and in the ionosphere. This precedence concerning the appearance of critical dynamics in the ULF magnetic field variations as compared to the appearance of criticality in the lower ionosphere could imply that the mechanism producing the ULF magnetic field anomaly drives the mechanism producing the VLF subionospheric propagation anomaly. This might be of great importance in studying the mechanism of LAI coupling. However, a further investigation of this issue is considered out of the scope of this paper and will be pursued in the future.

As it is well known, magnetosphere’s condition influences ionosphere. Geomagnetic disturbances such as storms, sudden commencements etc. highly influence ionosphere. On the other hand, a new interpretation on the relation between Kp index and EQs has been suggested in recent studies [54,55]. The planetary Kp index is obtained from a number of magnetometer stations at mid-latitudes and reflects global geomagnetic activity. Moreover, the main indices employed for the monitoring of magnetic effects, Kp, Dst, and AE, correlate rather well during periods of noticeable disturbances. During the analyzed period, 20 March 2016–15 April 2016, there were no noticeable magnetic field disturbances (max three-hourly Kp reached the value 6- on 7 April 2016), although there were variations with relative increase of Kp during specific periods as well as sudden commencements (http://www.gfz-potsdam.de/en/kp-index). It is not easy to attempt any correlation of the obtained NT analysis results with the variation of Kp, however it would be very interesting a direct application of the NT analysis to the Kp data and a comparison with the ionospheric NT analysis results in a future study.

## 6. Conclusions

The results of the first attempt of applying the NT criticality analysis to the subionospheric VLF data have been presented in the present article. As summarized in Section 4, the lower ionosphere as seen by VLF propagation has exhibited critical characteristics from two weeks up to two days before the main shock of the disastrous 2016 Kumamoto EQs (15 April 2016). We note that four of the receiving stations, which are all situated on the east (Pacific) coast of Japan, showed either marginal or clear indications of criticality from 28 March 2016 up to 1–2 April 2016. However, it may be possible that this behavior is related to the M5.9 EQ which happened in the Pacific Ocean coast of Japan on 1 April 2016, and not to the 2016 Kumamoto EQs.

Importantly, the NT analysis findings of criticality in this paper are found to be in extremely good consistency with the results of the conventional nighttime fluctuation method obtained for the same dataset and the theoretical calculations by means of the wave-hop method. Specifically, the time period for which VLF subionospheric propagation anomalies were identified by the conventional nighttime fluctuation method overlaps with the criticality dates revealed by the NT analysis method, while the spatio-temporal evolution of the ionospheric perturbation associated with the Kumamoto EQs obtained by the wave-hop method matches the progressive appearance of critical dynamics in the studied receivers.

We have also observed that the appearance of critical dynamics in the ground-observed ULF magnetic field recorded at a magnetic observatory close to the Kumamoto EQs epicenters precedes the appearance of criticality in the lower ionosphere. This could imply that the mechanism producing the ULF magnetic field anomaly drives the mechanism producing the VLF subionospheric propagation anomaly. However, more investigation is necessary. A multi-parameter analysis including the ULF/ELF radiation as a signature of atmospheric perturbation [56] is highly required in order to elucidate the mechanism of LAI coupling in the future. Moreover, it would be very interesting a direct application of the NT analysis to Kp data and a comparison with the ionospheric NT analysis results in a future study.

## Figures and Tables

**Table 1:** VLF/LF transmitters and receivers information.

Transmitter		Receiver	
Call Sign	Location (Frequency)	Call Sign	Location
JJI	Miyazaki (22.2 kHz)	AKT	Akita
JJY	Fukushima (40 kHz)	ANA	Anan
NLK	Seattle (24.8 kHz)	IMZ	Imizu
NPM	Hawaii (21.4 kHz)	KMK	Kamakura
NWC	W. Australia (19.8 kHz)	KTU	Katsuura
		NSB	Nakashibetsu
		STU	Suttsu
		TYH	Toyohashi

**Table 2:** NT Analysis results for the paths between all eight VLF/LF receivers and JJI transmitter. Only the receiver data which presented criticality are mentioned. Bold fonts indicate clear criticality indications, while normal fonts denote marginal indications (e.g., cases for which criticality is found for a few threshold values only, or for which the NT analysis parameters ($\kappa_{1}$, $S_{nt}$, $S_{nt-}$ and 〈D〉) marginally satisfy criticality criteria).

Date	NF	TR	DP
28 March 2016		TYH	ANA
29 March 2016	KMK, TYH		ANA
30 March 2016			ANA
31 March 2016	**KTU**		
1 April 2016	**KTU**	**KTU, KMK, TYH**	
2 April 2016		**KTU**	
3 April 2016			
4 April 2016			
5 April 2016			**KTU**, AKT
6 April 2016	NSB	NSB	**KTU**, ANA
7 April 2016	NSB		**KTU**, NSB
8 April 2016		**STU**	**STU**, NSB
9 April 2016	**KTU**	**STU**	**STU, IMZ**
10 April 2016	**AKT**	**STU**	**KMK, STU**
11 April 2016	**IMZ**	**KTU, STU**, IMZ	**KMK, STU**
12 April 2016	**IMZ**	**KTU, AKT**, IMZ	**KMK**, AKT
13 April 2016		**KTU, AKT**	
14 April 2016			
15 April 2016	KMK	KMK	
